# Response of Laying Hens to Repletion and Depletion in Dietary Balanced Protein

**DOI:** 10.3390/ani12192567

**Published:** 2022-09-26

**Authors:** Ingryd Palloma Teodósio da Nóbrega, Matheus de Paula Reis, Rony Riveros Lizana, Thaila Fernanda de Moura, Guilherme Ferreira da Silva Teofilo, Letícia Cardoso Bittencourt, Nilva Kazue Sakomura

**Affiliations:** 1 Department of Animal Sciences, Faculty of Agrarian and Veterinary Science, São Paulo State University, Jaboticabal 14884-900, SP, Brazil; 2 DSM Brazil, Mairinque 18120-000, SP, Brazil

**Keywords:** egg production, ideal amino acid profile, egg components, body composition

## Abstract

**Simple Summary:**

Considering the dynamism of the price of feed ingredients, there may be an opportunity to reduce the egg production cost by reducing the nutritional value of the feed. In this situation, it is important to understand the consequences of a feed and the ability of laying hens to recover from a previously deficient diet. This study aimed to evaluate the responses of laying hens in two scenarios of dietary balanced protein, namely, repletion and depletion. In the repletion phase, laying hens were given low dietary balanced protein (BP) in the growing phase (8 to 18 w-old), which was then changed (19 to 102 w-old) to a high dietary BP. The depletion treatment follows the opposite logic. The laying hens were monitored from 8 to 102 w-old to collect information about performance, egg quality, and body composition. The low dietary balanced protein feed affected the age at first egg and body composition, but there was a performance recovery after 19 weeks of a repletion treatment (at 38 w-old). The opposite result was observed for laying hens consuming a depleted feed. The egg components were affected only by the feed given in the laying phase. The laying hens were able to overcome a reduction in dietary balanced protein once they were given an opportunity to do so; however, in this study, 19 weeks were necessary for laying hens to achieve a steady state.

**Abstract:**

This study was carried out to investigate the response of laying hens given a repletion or depletion in dietary balanced protein (BP) during the laying phase period. At the beginning of the rearing period (eight w-old), four-hundred pullets were equally distributed and received one of two experimental feeds: 1-Low BP (L) and 2-High BP (H). For the laying period (19 to 102 w-old), four feeding programs were designed based on the same treatments for rearing phases (LL, HH, LH, HL), where subsequent letters indicate the feed received during the rearing and laying period, respectively. The performance responses, egg quality, and body composition were periodically collected during the laying period. Two-way ANOVA repeated measures analysis was applied to evaluate the data. Nonlinear regression models with groups were used to compare treatments in the laying phase, with the treatments being the group evaluated. All performance traits were somehow influenced by the level of BP in the feed (*p* < 0.050). Hens subjected to the repletion treatment (LH) demonstrated a recovery in performance after 38 w-old. The opposite result was observed for hens on the depletion treatment (HL). All egg components were affected by dietary BP (*p* < 0.050). Laying hens demonstrated a limited capacity to overcome a reduction in dietary BP during production, but they were able to recover from a previous deficient feed once they were given an opportunity to do so.

## 1. Introduction

The growing period of a laying hen is the most critical time in a hen’s life and the mistakes made during this period are difficult to rectify [1]. Many factors, e.g., quantitative or qualitative feed restriction [2,3], feed program [4,5], and nutritional imbalance [4,6], in the starter, grower, and/or developer phases were reported to affect the growth curve, early egg weight, and sexual maturity of pullets and, consequently, egg production. Therefore, the maximum genetic potential can only be achieved when the bird is provided with all its nutritional requirements [7,8], especially when the objective is to extend the productive life of laying hens.

During the pullet-rearing period, the focus is mainly on managing pullet body weight and body weight uniformity. However, current pullet feeding programs can lead to pullets of similar body weight but with markedly different body compositions, which may affect life production [9]. Advances in genetic selection produced pullets quite different to those from only a few years ago. In the literature, there are reports regarding nutritional recommendations for egg-type hens during the rearing and laying period [10,11]; however, information is still needed on combined feeding strategies between both periods in modern lines of hens. The effects of dietary balanced protein for hens in the rearing phase and its impact on the long-term laying cycle were not investigated so far.

The ideal supply of digestible amino acids during pullet formation is essential to ensure the growth of organs, muscles, and the skeleton [12], while in the productive period, this contribution is essential for body maintenance and for egg component development [13]. Thus, the lack of balance of essential amino acids in the diet can affect pullet formation and its performance in the laying phase [14,15]. In this context, we hypothesize that balanced protein levels affect pullet formation, leading to a shift in the long-term laying cycle, and the repletion in dietary balanced protein may recover the responses of laying hens; therefore, the aim of the present research is to evaluate the impact of depletion and repletion in dietary balanced protein on body composition, performance, and egg quality in laying hens submitted to low and high dietary protein during the rearing period.

## 2. Materials and Methods

### 2.1. Ethics Approval

All procedures described were approved by the Ethical Committee on the Use of Animals of the School of Agrarian and Veterinary Sciences, São Paulo State University (UNESP), Jaboticabal, São Paulo, Brazil (Process 012598/2018); approved on 14 February 2019.

### 2.2. Birds, Husbandry, and Experimental Design

Four hundred Lohmann LITE LSL-NA were obtained from a local commercial facility (Planalto Postura LTDA) at one day old and raised in conventional cages according to genetic guideline recommendations prior to the beginning of the trial. At eight weeks of age, pullets were moved to wire-rearing cages (375 cm^2^ per pullet) and moved again at 19 weeks of age to wire-laying cages (563 cm^2^ per hen). Each cage was equipped with a feeder and nipple drinker. Temperature, humidity, and lighting were maintained according to the recommendation of the Lohmann LSL-NA Management Manual (Lohmann Tierzucht GmbH, Cuxhaven, Germany).

At the start of the trial (eight weeks of age), 400 pullets were individually weighed (0.592 ± 0.012 kg) and moved to 20 cages to which two treatments (low and high dietary balanced protein) were randomly assigned, performing 10 replicates of 20 pullets each. At 19 weeks of age, each treatment was separated in two (1-low, 2-high, 3-repletion, and 4-depletion in dietary balanced protein), giving a total of four treatments randomly distributed in five replicates each. During each experimental period, water and feed were provided ad libitum. The lighting program was set at 24 h light at the first week, reduced gradually to 12 h light and 12 h dark up to 10 weeks of age, and maintained until the pullets achieved 5% of egg production (20 weeks of age). After the onset of egg production, the lighting program was gradually increased from 12 to 16 h of light and kept constant up to 102 weeks of age.

A three-phase feeding program was used in the rearing period: grower (8–11 w-old), developer (12–15 w-old), and pre-layer (16–18 w-old); while a five-phase feeding program was used for the laying period: Layer 1 (19 to 26 w-old), Layer 2 (27 to 46 w-old), Layer 3 (47 to 66 w-old), Layer 4 (67 to 82 w-old), and Layer 5 (83 to 102 w-old).

### 2.3. Experimental Feeds

Experimental feeds consisted of two levels of dietary balanced protein, herein named low (L) and high (H). Dietary balanced protein was defined as a constant ratio of essential amino acids with lysine [16], and the ratio was the same proposed by the breeding company (Lohmann Tierzucht GmbH, Cuxhaven, Germany). Standardized ileal digestible lysine (SID-Lys) was used as a reference to produce the two levels of dietary balanced protein. The remaining nutrients and energy in the feed were as recommended by the guideline [10].

The grower, developer, and pre-layer feeds contained, respectively, 0.65, 0.56, and 0.59% of SID-Lys for L feeds and 0.96, 0.84, and 0.89% of SID-Lys for H feeds (Table 1).

In the laying period (from 19 to 102 weeks of age), half replications continued receiving the L or H dietary balanced protein feeds (LL and HH), and the other half was submitted to repletion (LH) or depletion (HL), where subsequent letters indicate the feed supplied on rearing and laying phases, respectively. In the layer period, each one of the five feeds contained, respectively, 0.54, 0.52, 0,50, 0.48, and 0.46% of SID-Lys for L feeds and 0.82, 0.79, 0.75, 0.73, and 0.70% of SID-Lys for H feeds (Table 2).

### 2.4. Performance Data

In the rearing period, cumulated feed intake and body weight were determined at 18 weeks of age. Mortality was registered daily and used to correct the feed intake. During the laying phase, egg production and mortality were recorded daily. Once a week, all eggs produced in one day were weighed and the egg mass was calculated. Feed intake was determined fortnightly and corrected for mortality. The age at sexual maturity was determined for each experimental unit and was defined as the age at 50% of egg production. Hen-housed egg production was calculated as the total number of eggs produced per number of housed hens at 19 w-old.

### 2.5. Body Composition

Laying hens selected at the beginning of the trial were individually scanned using dual-energy X-ray absorptiometry (DXA, Hologic-QDR^®^ model 13.4.2., Marlborough, MA, USA). In the rearing phase, DXA measurement was performed on the last day of each feeding phase, whereas in the laying phase, measurements were taken every 28 days. The same birds were scanned over time. For that, a total of 16 pullets per treatment were used in the rearing phase and 8 hens per treatment in the laying phase. The same hens were scanned over time. Prior to each scan, hens were fasted for five hours, weighed, anesthetized with isoflurane (2%) diluted in 100% of oxygen, and positioned in dorsal decubitus with the wings and legs flexed [17]. The fat mass (g), lean mass (water + protein content, g), bone mineral content (g), and bone mineral density (g/cm^2^) were registered. Alves et al. [17] equations were used to estimate the ash, fat, and protein content, as described below:

(1) $$Ash_{\%}=\frac{e^{0.44045xln\left(BMC\right)+0.33779xln\left(lean\right)}}{BW}\times 100$$

(2) $$Fat_{\%}=\frac{e^{-4.351-0.14257xln\left(BMC\right)+0.19065xln\left(Fat\right)-1.74711xln\left(Lean\right)+2.94089xln\left(BW\right)}}{BW}\times 100$$

(3) $$Protein_{\%}=\frac{e^{-2.007+1.06xln\left(lean\right)}}{BW}\times 100$$

where e is Euler’s number, ln is the natural logarithm, BMC is the bone mineral content (g), lean is the lean mass (g), Fat is the fat mass (g), and BW is the body weight (g), obtained by DXA.

### 2.6. Egg Traits and Egg Components Measurement

Every four weeks, three eggs per experimental unit were sampled in three sequential days, totaling nine eggs per experimental unit. The eggs were individually weighed and numbered. The egg components, albumen, yolk, and dry eggshell were measured. Before measurement, the eggshell was washed with tap water and dried using a forced oven at 55 °C for 24 h. Additionally, the strength and shell thickness were analyzed using the Nabel Digital Egg Tester 6000^®^ (Kyoto, Japan).

### 2.7. Statistical Analysis

The feed intake, body weight, and body composition measured during the rearing period were analyzed with one-way ANOVA, using a generalized linear model. In the laying phase, the age at sexual maturity and hen-housed egg production were evaluated with one-way ANOVA, and other responses were evaluated as a two-factor repeated measure to determine the effects of dietary treatments over time, using a mixed model. Fixed factors are represented by the four treatment groups (LL, LH, HH, and HL) and the age of the hens, whereas the experimental unit represents the random factor. The data were analyzed considering 21 cycles of 4 weeks each. Orthogonal contrasts were elaborated to investigate the effects of repletion (LL vs. LH) and depletion (HH vs. HL) in dietary balanced protein. Differences were considered to be significant at a probability of 5%. The Statistical Analysis System (SAS Institute Inc., Cary, NC, USA) was used to perform both a one-way ANOVA and the two-factor repeated measure analysis procedures.

To investigate how the responses differed between treatment groups over time, non-linear regression with groups was used, the groups being the dietary balanced protein [18]. The average data per replicate were treated as the experimental unit. Two exponential models were applied and those with the lower Akaike information criterion value [19], were used to describe the response variable in function of age (weeks). The models used were:

(4) $$\mathrm{Linear}\;\mathrm{plus}\;\mathrm{exponential}\text{:}\;y=A+B\times \left(R^{age}\right)+C\times age$$

where A and C are the y-intercept and slope of the linear segment, respectively, B is the y-intercept of the exponential segment, and R is the exponential base.

(5) $$\mathrm{Exponential}\text{:}\;y=A1+B1\times \left(R1^{age}\right)$$

where A1+B1 is the y-intercept, and R1 is the exponential base.

## 3. Results

### 3.1. Rearing Period

The performance parameters and body composition of pullets during the rearing phase are shown in Table 3. The dietary balanced protein affected the body weight (*p* = 0.009) but had no effect on cumulated feed intake (*p* = 0.34). Even with a similar cumulated feed intake between groups, pullets consuming the feed with a higher level of balanced protein were about 3% heavier at the end of the rearing period (18 weeks of age). The observed results indicate that pullets fed with the L feed were not able to consume a sufficient amount of protein to support their growth, reducing the body weight gain during the rearing period. The dietary balanced protein did not affect the body composition evaluated (*p* = 0.96, *p* = 0.17, and 0.54, respectively, for ash, fat, and protein).

### 3.2. Laying Period

#### 3.2.1. Performance

The age at sexual maturity was statistically different between groups (Table 4). Increasing the dietary balanced protein about one week before the onset of lay did not change the age at sexual maturity (LL vs. LH, *p* = 0.51), and a similar response was observed when a decrease in dietary balanced protein was applied (HH vs. HL, *p* = 0.23). On the other hand, the feed offered in the rearing phase influenced the age at sexual maturity (*p* < 0.001). Laying hens consuming the L feed had seven days of delay in the age at sexual maturity in comparison with hens consuming the H feed. Similarly, the reduction in dietary balanced protein in the rearing period affected the hen-housed egg production (*p* = 0.003). On average, laying hens given the L feed in the rearing phase reduced the egg production by 30 units in comparison with hens consuming the H feed; however, the repletion or depletion in dietary balanced protein did not affect this response variable (*p* = 0.21 and *p* = 0.33, respectively, for repletion and depletion in dietary balanced protein).

The interactions for the two-way repeated measure were significant for all performance responses evaluated in the laying period (Table 5). As a consequence of the feed given in the previous phase, feed intake was different between groups during the first six weeks after the onset of lay. When laying hens consumed the H feed in the rearing period, they increased their feed intake in a higher ratio compared with hens from the L group (Table S1). This difference, however, was not consistent over time and the mean feed intake accounted for the whole laying phase was similar between groups.

**Figure 1:**
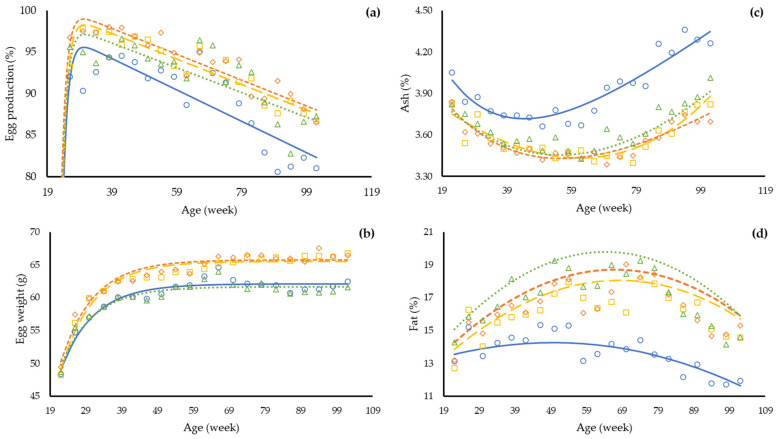
Observed and predicted egg production (**a**), egg weight (**b**), body ash (**c**), and body fat (**d**) of laying hens from 19 to 102 weeks old in response to four diet programs with distinct levels of dietary balanced protein feeds. Treatments: LL (**○**, **—**) and HH (**◊**, **- -**), reduction and accretion in using balanced protein (BP) during rearing period and laying period; LH (**□**, **– –**) and HL (**∆**, **∙∙∙**), repletion and depletion in BP in laying period.

**Figure 2:**
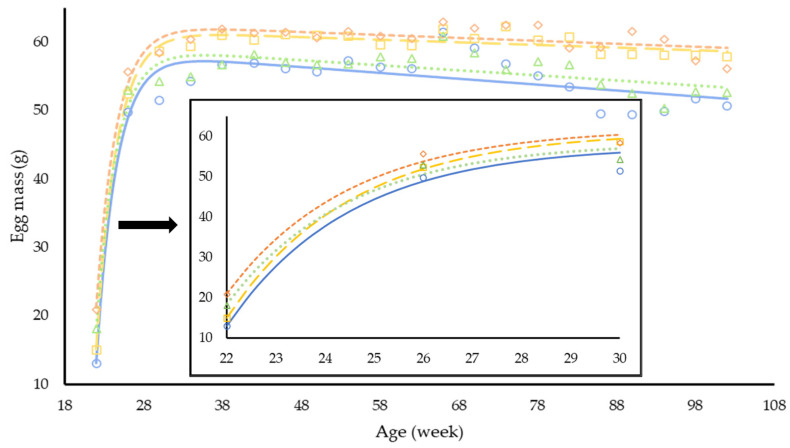
Observed and predicted egg mass (g) of laying hens from 19 to 102 weeks of age in response to levels of dietary balanced protein. Treatments: LL (**○**, **—**) and HH (**◊**, **- -**) are low and high dietary balanced protein treatments, respectively; LH (**□**, **– –**) and HL (**∆**, **∙∙∙**) are the repletion and depletion in dietary BP applied to laying hens from 19 to 102 weeks of age.

The non-linear regression with groups applied for feed intake in function of age (Table 6) indicates that three parameters were affected by treatment, and only the parameter R was similar between groups. On average, the repletion in dietary balanced protein (LL vs. LH) improved egg production (3.7%, *p* < 0.001), egg weight (3.0 g, *p* < 0.011), egg mass (4.9 g, *p* < 0.001), feed conversion (0.16 g/g, *p* < 0.001), and increased mean body weight (153 g, *p* = 0.011). A depletion in dietary balanced protein (HH vs. HL) reduced egg weight (−3.5 g, *p* < 0.001) and egg mass (−4.5 g, *p* < 0.001) and increased feed conversion (0.18 g/g, *p* < 0.001) in the laying hens. The regression with groups (Table 6) demonstrates that the performance responses of the laying hens were affected by treatment, and only the exponential base (R) of the equation was similar for all treatments indicating a similar behavior between groups but different ratios and maximum/minimum estimates. The peak of egg production was estimated at 30 weeks of age for all treatment groups; however, the repletion in dietary balanced protein seemed to recover the egg production rate at the peak (98%) in comparison with hens in the LL group (95%) (Figure 1). The LH group reached the peak of egg mass two weeks before the LL group and produced 4 g more egg mass at the peak. (Figure 2). The depletion in balanced protein (HH and HL) affected the peak of egg mass for approximately one week. These results can also be observed in the Table S2.

#### 3.2.2. Body Composition

There was an interaction between treatment and the age of laying hens for ash (*p* < 0.001), fat (*p* < 0.001), and protein (*p* = 0.009) contents in the body (Table 5 and Table S3). The results suggest that the differences are mainly due to the group of hens fed with the LL feeds (Figure 2). On average, hens given a repletion in dietary balanced protein increased 2.4 percentual points in the fat content (*p* = 0.007) compared with hens in the LL group but reduced by 0.36 and 0.70 percentual points in the contents of ash (*p* < 0.001) and protein (*p* = 0.014) in the body. The regression between groups indicates that both dietary balanced protein and the repletion/depletion treatments affect the dynamics of body composition over time (*p* < 0.001, Table 7).

#### 3.2.3. Egg Quality

Overall, the interaction between treatment and the age of hens was statistically different for all egg components and eggshell strength (Table 5, Tables S4 and S5). Laying hens consuming the LH and HH feed produced eggs with a heavier yolk in comparison with hens consuming the LL or HL feeds (around a 6% difference). The results indicate that the feed given in the rearing phase has a limited influence on the yolk production. The differences observed for albumen and eggshell weights suggest a similar behavior. The heavier albumen and eggshell were produced by hens consuming a feed with a higher balanced protein level (*p* = 0.003 and *p* = 0.001, respectively), disregarding the feed given in the rearing phase. The differences in eggshell strength were evident for the depleted treatment (HH vs. HL, *p* = 0.048), reducing the eggshell strength by about 3%. The eggshell thickness was similar between treatments (LL vs. LH, *p* = 0.87 and HH vs. HL, *p* = 0.35). The regression in groups demonstrates that individual equations are necessary to predict the egg components and eggshell strength over time (Table 8).

## 4. Discussion

The nutrition given to laying hens in the rearing phase may influence the growth and, consequently, their degree of body maturity. However, it is well known that sexual maturity is most influenced by the photoperiod, with body weight having a minor effect [20], opening an opportunity to change the pullet’s nutrition without affecting the sexual maturity, but the hens’ response over long-term egg production needs to be investigated. The objective herein was to evaluate laying hens regarding the effect of dietary balanced protein given in the rearing phase and how they respond to a repletion or depletion in dietary balanced protein in the laying phase. We hypothesized that offering a low dietary balanced protein feed to pullets from 8 to 18 weeks of age would produce a lighter hen with, perhaps, higher body fat content when compared with a hen consuming a high balanced protein feed. Those differences would have a minimum impact on the age at sexual maturity, but the low dietary balanced protein feed would not be sufficient to sustain a high egg production or egg mass. An even more interesting question to be answered is whether those effects are reversible if the dietary balanced protein is repleted in the laying phase.

In this study, the age at sexual maturity (50% of egg production) was influenced by the feed given in the rearing phase, where pullets in the higher dietary balanced protein feed reached sexual maturity approximately 7 days before, which may have elicited an increase in feed intake prior to the laying hens consuming the L feed, minimizing the difference in cumulated feed intake at the end of the rearing phase (18 weeks of age). In fact, the results published elsewhere by Da Nóbrega et al. [21] demonstrate an increase in feed intake due to a reduction in dietary balanced protein, which is minimized when pullets approach 15 weeks of age. According to Bendezu et al. [22], the development of the ovary and oviduct is maximized around 15 to 16 weeks of age, which affects the needs for energy and nutrients and, consequently, feed intake. Body weight, on the other hand, was clearly affected by dietary balanced protein, with no effect on body composition. Those results indicate that pullets from distinct groups were at a different degree of body maturity, which may also influence the age at sexual maturity. Lewis and Morris [20] found evidence that laying hens maintained in the same photoperiod but with different body weights achieved the onset of lay and the age at sexual maturity on different days, corroborating our observations. However, the aforementioned authors highlighted that the photoperiod has much more influence on the onset of lay than body weight.

The rate of sexual maturation is coordinated by hormones such as the luteinizing hormone (LH) and follicle-stimulating hormone (FSH), produced in the pituitary gland [23]. The release of LH and FSH is stimulated by the gonadotropin-releasing hormone (GnRH), produced in the hypothalamus [24], also referred to as the extra-retinal or deep encephalic photoreceptor, since light perceived in this region of the brain will control the secretion of GnRH. A system called the hypothalamo–hypophyseal–gonadal axis allows the GnRH to reach the pituitary gland and initiate the release of LH and FSH [23]. Another hormone that also controls the LH and FSH release is the gonadotropin inhibitory hormone (GnIH). The GnIH, also produced in the hypothalamus, is antagonistic to GnRH and will prevent the pituitary from releasing the LH and FSH hormones [25]. Both GnRH and GnIH are peptide hormones, thus, requiring a receptor in the site of action to bring about its function. The GnIH receptors in the pituitary are reported to decrease in Lohmann hens between 17 to 20 weeks of age, while the GnRH receptors increase at the same age [26]. Those events may increase the release of LH and FSH and contribute to the onset of lay. A possible explanation for the shift in the age at sexual maturity for hens submitted to the same photostimulation but consuming different levels of dietary balanced protein is that a delay in the degree of body maturity observed in hens consuming the L feed may also delay the changes in GnRH and GnIH receptors in the pituitary gland, but this hypothesis needs to be tested.

The objective to produce different laying hens at the end of the rearing phase was achieved, but the body composition was similar between groups. The effect of dietary balanced protein over body fat is well documented in the literature for broilers and breeders [27,28]. Those studies report that body fat percentage increases with the reduction in dietary balanced protein. Our results demonstrate that body fat content was similar between treatment groups at the end of the rearing phase, showing a different trend from the ones reported for broilers and breeders. One may expect that reducing dietary balanced protein would reduce the amount of protein available for deposition, and hence, the energy once used for protein deposition would be available for lipid deposition. These events become especially true if the feed intake is constant or increases with the reduction in dietary balanced protein. However, our observations suggest that this is not the case for growing pullets after eight weeks of age. The degree of body maturity may, again, be one possible explanation. The reduction in dietary balanced protein delays the body protein deposition and, perhaps, the development of reproductive organs in laying hens. As the hens approach their sexual maturity, the development ratio of the ovary and oviduct increases rapidly, and lipid deposition in the ovary contributes mostly to such an increase [22]. Since laying hens in the H group were advanced in body development, their ovary and oviduct development may have started earlier when compared with hens in the L group, increasing the lipid deposition in the body and minimizing the differences from pullets consuming the L feed.

Once in production, it is useful to know if the consequences of giving a low protein feed in the rearing phase can be reversed. For that, a repletion treatment was included in the treatment design. The overall results demonstrate that repleted hens (LH) increased all responses evaluated, with an exception for daily feed intake and eggshell traits. The data presented (Table 5) demonstrate that repletion in dietary balanced protein could be a strategy to recover a pullet that reaches sexual maturity with low body weight. In addition, there may be an economic benefit to reduce balanced protein in the feed because the feed price would decrease [27]. Since feed intake was similar between groups, the feeding cost (feed intake x feed price) would also reduce. The egg mass was similar between hens consuming the H feed in the laying phase (LH and HH), suggesting that the revenue obtained from either group of hens would be the same. Nevertheless, an economic investigation is necessary to better understand this issue, which was not the goal of this study. Another issue that is worth investigating is related to the effects of depletion in dietary balanced protein. The change in the price of feed ingredients may trigger nutritionists to reduce the price of a feed formula, sometimes by reducing the dietary balanced protein level. To properly evaluate the laying hens’ response due to a reduction in dietary balanced protein, the current status of the bird needs to be accounted for.

We showed herein that laying hens receiving a high dietary balanced protein feed in the rearing phase were able to increase feed intake at the beginning of egg production when dietary balanced protein was depleted. As a result, this group of birds had the highest lipid content in the body, even though, on average, such a difference was not statistically different from hens in the HH group, possibly because to recover the amount of dietary balanced protein that was removed from the feed, laying hens would need to increase their feed intake approximately 40%, which was, perhaps, beyond the intestinal bulk capacity of these hens. In this study, to reduce dietary balanced protein, it was necessary to include more wheat bran in the feed compared with other treatments. That might have limited the bulk capacity of the gastrointestinal tract, constraining the feed intake. Recently, Nascimento et al. [29] demonstrated that broiler breeders could increase their feed intake as the feed was diluted to achieve their nutrients and energy needs, but the intake of feed decreased at a higher dilution.

An interesting piece of data produced in this study is the time necessary to change the response of laying hens when a repletion or a depletion feed is offered. According to the repeated measures analysis, it took 11 weeks to detect a difference in egg production between groups, while for egg weight and egg mass, seven weeks after the beginning of the repletion and depletion treatments were necessary to affect those variables. The nonlinear regression also indicates that the ratio of increase for each mentioned variable was different, which is demonstrated in Figure 2. A decrease in egg production, egg weight, and egg mass was reported in laying hens consuming crescent levels of dietary balanced protein from 26 to 77 weeks of age [30]. The pattern of body chemical components over time changed consistently after 50 weeks of age, especially for body fat. Laying hens in the LL group demonstrated the lowest body fat content compared to the other treatments. The reduction in body fat content for laying hens consuming a low dietary balanced protein feed was not expected; however, Kumar et al. [31] found a quadratic response in abdominal fat in function of dietary balanced protein concentration.

In the present study, the results from repletion and depletion groups might require a separate interpretation. When compared with hens from the LL group, the higher value of body fat content observed in repleted hens might be related to the lipid content in the ovarium, since those hens produced eggs with heavier yolk. On the other hand, compared with the HH group, depleted hens increased body fat deposition, possibly due to an increase in feed intake during the first weeks after the depleted feed was offered, increasing energy intake. In either situation, any conclusion over the dynamics of body fat content in laying hens should be carefully evaluated, and more studies are necessary to better elucidate this response.

In this study, the results observed for egg production and egg weight suggest that the feed offered in the rearing phase has little influence on those responses. The hen-housed egg production, however, was influenced only by the feed offered in the rearing phase (LL + LH vs. HH + HL). The observed differences might be a consequence of the viability observed during the trial. The viability of laying hens consuming the L feed during the rearing phase was 87.5%, whereas hens consuming the H feed had a viability of 90%. Grossman et al. [32] suggest that hens with similar rates of egg production may have different egg production curves, mainly due to persistency. The persistency in egg production is defined as the decline ratio observed over time [33,34]. In this study, the parameter C in the equation adjusted for egg production in function of time is related to a declining ratio after the maximum point (peak of egg production). The results indicate that laying hens in the LL group reduced their egg production after the peak faster than the other groups, followed by HH, LH, and HL.

## 5. Conclusions

The results presented herein demonstrate how pullets respond to dietary balanced protein and the consequences of a repletion or a depletion in dietary balanced protein in the laying phase. The adverse effects of reducing the balanced protein in the growing phase were minimized by repleting the dietary balanced protein in the laying period. On the other hand, depletion in the balanced protein in the layer phase reduced the performance of hens, reaching similar results to hens consuming the lower protein diet during the whole study.

## Figures and Tables

**Table 1:** Composition and calculated nutritional content of low and high dietary balanced protein feeds in the rearing period.

	8–11 Weeks		12–15 Weeks		16–18 Weeks	
Ingredients	Low	High	Low	High	Low	High
Corn (7.8%)	68.0	56.3	63.0	53.4	61.0	49.9
Soybean meal (45%)	15.0	31.6	10.0	23.8	10.0	24.2
Wheat bran	13.0	7.10	20.0	15.0	20.0	14.0
Potassium carbonate	0.240	-	0.210	-	0.280	0.005
Corn gluten (60%)	-	-	1.50	1.50	-	2.00
Meat and bone meal 48%	-	-	2.66	2.66	2.97	3.87
Soy oil	0.150	1.50	0.370	1.50	0.685	1.42
Dicalcium phosphate	1.17	1.20	0.140	0.161	0.210	-
Limestone	1.46	1.32	1.38	1.26	3.98	3.75
Salt	0.287	0.420	0.215	0.306	0.215	0.256
Sodium bicarbonate	0.200	-	0.200	0.065	0.198	0.110
Vit. and min. supplement ^1^	0.200	0.200	0.200	0.200	0.200	0.200
DL-Methionine (99%)	0.055	0.161	-	0.114	0.045	0.111
L-Lysine HCl (78%)	0.100	0.024	0.038	-	0.095	0.027
L-Threonine (98.5%)	-	0.031	-	-	-	-
Choline chloride (60%)	0.100	0.100	0.100	0.100	0.100	0.100
Total	100	100	100	100	100	100
Calculated nutritional content (%)						
AMEn (kcal/kg) ^2^	2883	2880	2860	2860	2778	2778
Crude protein ^3^	14.3	20.0	14.7	19.5	13.9	20.2
Dig. Lysine	0.645	0.960	0.560	0.840	0.593	0.890
Dig. Methionine + cysteine	0.476	0.709	0.434	0.653	0.442	0.662
Dig. Threonine	0.475	0.703	0.471	0.635	0.441	0.652
Dig. Tryptophan	0.148	0.226	0.139	0.203	0.133	0.206
Dig. Isoleucine	0.496	0.760	0.480	0.698	0.444	0.719
Dig. Valine	0.568	0.827	0.573	0.787	0.532	0.812
Calcium	1.04	1.04	1.05	1.05	2.08	2.08
Available Phosphorus	0.460	0.460	0.430	0.430	0.457	0.457
Sodium	0.180	0.180	0.170	0.170	0.170	0.170

^1^ Inclusion of 2 kg of premix/kg of feed. Content/kg of premix: Vit. A 4,850,00 Ul, Vit. D3 1,350,000 Ul, Vit. E 8500 Ul, Vit. K3 1395 mg, Vit. B1 1000 mg, Vit. B2 2570 mg, Pantothenic acid 5295 mg, Vit. B6 1525 mg, Vit. B12 7500 mcg, Niacin 19.45 g, Folic acid 500 mg, Biotin 41.50 mg, Choline chloride 75 g, Iron 22 g, Copper 4500 mg, Manganese 25 g, Zinc 25 g, Iodine 500 mg, Selenium 125 mg, Phytase 300,000 FYT. ^2^ Nitrogen-corrected apparent metabolizable energy. ^3^ Values represent the mean analyzed composition by near-infrared spectroscopy (NIR).

**Table 2:** Composition and calculated nutritional content of low and high dietary balanced protein feeds in the laying phase.

	19–26 Weeks		27–46 Weeks		47–66 Weeks		67–82 Weeks		83–102 Weeks	
Ingredients	Low	High	Low	High	Low	High	Low	High	Low	High
Corn (7.8%)	65.0	53.0	67.0	55.3	67.5	56.2	68.5	58.0	68.7	58.1
Soybean meal (45%)	10.1	22.0	13.0	23.9	11.6	22.3	11.7	21.4	11.9	20.2
Wheat bran	6.00	1.49	4.70	-	5.00	-	5.00	-	5.00	-
Potassium carbonate	0.560	0.340	0.470	0.302	0.525	0.325	0.500	0.315	0.510	0.340
Corn gluten (60%)	5.00	10.0	2.55	8.30	2.95	8.56	1.95	8.07	1.10	7.98
Soy oil	0.890	1.25	0.610	0.840	0.570	0.790	0.580	0.552	0.660	0.680
Dicalcium phosphate	1.30	1.32	1.14	1.17	1.09	1.14	1.09	1.14	0.98	1.04
Limestone	9.17	9.06	9.51	9.41	9.82	9.72	9.82	9.72	10.4	10.3
Salt	0.279	0.336	0.290	0.356	0.275	0.310	0.260	0.280	0.280	0.270
Sodium bicarbonate	0.200	0.110	0.183	0.080	0.168	0.110	0.190	0.157	0.160	0.172
Vit. and min. supplement ^1^	0.200	0.200	0.200	0.200	0.200	0.200	0.200	0.200	0.200	0.200
DL-Methionine (99%)	0.041	0.090	0.047	0.082	0.036	0.068	0.038	0.059	0.038	0.050
L-Lysine HCl (78%)	0.119	0.078	0.029	-	0.038	-	0.022	-	-	-
Choline chloride (60%)	0.100	0.100	0.100	0.100	0.100	0.100	0.100	0.100	0.100	0.100
Inert ^2^	1.023	0.621	0.150	-	0.159	0.171	0.052	-	-	0.495
Total	100	100	100	100	100	100	100	100	100	100
Calculated nutritional content (%)										
AMEn (kcal/kg) ^3^	2795	2795	2785	2785	2785	2785	2785	2785	2770	2770
Crude protein ^4^	15.6	21.4	14.6	20.6	13.3	20.2	11.7	18.7	11.4	18.7
Crude fiber	2.22	2.25	2.50	2.22	2.22	2.16	2.23	2.14	2.23	2.09
Dig. Lysine	0.544	0.816	0.524	0.786	0.500	0.750	0.484	0.726	0.464	0.696
Dig. Methionine + cysteine	0.480	0.720	0.464	0.696	0.448	0.672	0.432	0.648	0.416	0.624
Dig. Threonine	0.459	0.683	0.459	0.681	0.446	0.664	0.433	0.647	0.420	0.627
Dig. Tryptophan	0.123	0.192	0.131	0.196	0.124	0.188	0.123	0.183	0.121	0.176
Dig. Isoleucine	0.486	0.781	0.484	0.776	0.468	0.755	0.450	0.731	0.434	0.707
Dig. Valine	0.563	0.869	0.553	0.859	0.539	0.839	0.518	0.813	0.499	0.789
Calcium	3.95	3.95	4.05	4.05	4.15	4.15	4.15	4.15	4.35	4.35
Available Phosphorus	0.440	0.440	0.410	0.410	0.400	0.400	0.400	0.400	0.380	0.380
Sodium	0.175	0.175	0.175	0.175	0.165	0.165	0.165	0.165	0.165	0.165

^1^ Inclusion of 2 kg of premix/kg of feed. Content/kg of premix: Vit. A 4,850,00 Ul, Vit. D3 1,350,000 Ul, Vit. E 7785 Ul, Vit. K3 1195 mg, Vit. B1 1200 mg, Vit. B2 3000 mg, Pantothenic acid 4236 mg, Vit. B6 1522 mg, Vit. B12 7708 mcg, Niacin 16.21 g, Folic acid 500 mg, Biotin 41.50 mg, Choline chloride 93.75 g, Iron 22 g, Copper 4500 mg, Manganese 25 g, Zinc 25 g, Iodine 500 mg, Selenium 125 mg, Phytase 300,000 FYT. ^2^ Inert-Washed sand. ^3^ Nitrogen-corrected apparent metabolizable energy. ^4^ Values represent the mean analyzed composition by near-infrared spectroscopy (NIR).

**Table 3:** Performance and body composition of pullets at 18 weeks of age, fed two levels of balanced protein (BP).

	Treatments ^1^			
Variables	L	H	SEM ^2^	*p*-Value ^3^
Cumulative feed intake, g/bird	4445	4391	36.7	0.34
Body weight, g/bird	1203	1249	10.4	0.009
Ash, %	3.91	3.90	0.033	0.96
Fat, %	12.7	13.2	0.257	0.17
Protein, %	18.0	17.9	0.121	0.54

^1^ LL and HH, low and high dietary balanced protein (BP) during rearing period and laying period; LH and HL, repletion and depletion in dietary BP in laying period. ^2^ SEM: Standard error of the mean. ^3^ ANOVA at 5% probability level.

**Table 4:** Age at 50% egg production and hen-housed egg production of laying hens in response to dietary balanced protein levels in the development and laying periods.

Treatments ^1^	Age at 50% Egg Production	Hen-Housed Egg Production
LL	147	485
LH	146	500
HH	140	529
HL	142	517
SEM ^2^	1.10	8.11
*p*-value	<0.001	0.003
Orthogonal Contrasts		
LL vs. LH	0.51	0.21
HH vs. HL	0.23	0.33
(LL, LH) vs. (HH, HL)	<0.0001	<0.001

^1^ LL and HH, low and high dietary balanced protein (BP) during rearing period and laying period; LH and HL, repletion and depletion in dietary BP in laying period. ^2^ SEM: Standard error of the mean.

**Table 5:** Analysis of variance and contrasts for performance, body composition, and egg quality of laying hens from 19 to 102 weeks old in response to levels of dietary balanced protein (LL or HH), repleted (LH) and depleted feeds (HL).

	Treatments					Source of Variation			Orthogonal Contrasts	
Variables	LL	LH	HH	HL	SEM ^1^	Cycles ^2^	Treatments	Interaction	LL vs. LH	HH vs. HL
Performance										
Feed intake, g/bird/day	107	107	108	109	1.56	***	n.s.	*	n.s.	n.s.
Egg production, %	86.3	90.0	91.6	89.7	1.33	***	***	***	**	†
Egg weight, g	60.3	63.3	63.6	60.1	0.619	***	***	***	***	***
Egg mass, g	52.4	57.3	58.4	53.9	1.01	***	***	***	***	***
Feed conversion ratio, g/g	2.17	2.01	1.90	2.08	0.029	***	***	***	***	***
Mean body weight, g/hen	1422	1575	1658	1615	41.2	***	**	***	*	n.s.
Mean Body Composition (%)										
Ash	3.93	3.57	3.55	3.65	0.073	***	***	***	***	n.s.
Fat	13.7	16.1	16.4	17.0	0.736	***	**	***	**	n.s.
Protein	18.1	17.4	17.5	17.0	0.263	***	**	**	*	†
Mean Egg Response										
Yolk, g	15.9	16.8	17.1	15.9	0.250	***	***	***	**	***
Shell, g	5.86	6.04	6.12	5.83	0.082	***	**	***	*	***
Albumen, g	39.2	41.0	40.9	38.9	0.548	***	**	***	**	**
Shell strength, kgf	4.35	4.43	4.47	4.33	0.101	***	n.s.	*	n.s.	*
Shell thickness, mm	0.381	0.380	0.383	0.380	0.004	***	n.s.	†	n.s.	n.s.

^1^ SEM: Standard error of the mean. ^2^ Cycles: Every 4 weeks from 19 to 102 weeks of age. * *p* ≤ 0.05, ** *p* ≤ 0.01, *** *p* ≤ 0.001, † *p* ≤ 0.10, and n.s. *p* > 0.10.

**Table 6:** Regression models to estimate the performance of laying hens from 19 to 102 weeks old consuming levels of dietary balanced protein (LL or HH), repleted (LH) and depleted feeds (HL).

Parameters	LL	LH	HH	HL
	Feed Intake, g/bird/day			
A	109.90	110.30	113.20	111.60
B	−2786	−2642	−2541	−2231
C	0.0020	−0.0014	−0.0376	−0.0110
R	0.8185			
SEM ^1^	4.04			
R2 ^2^	75.0			
	Egg Production, %			
A	101.60	103.20	103.90	101.90
B	−159,459,287	−159,945,455	−123,767,487	−138,501,947
C	−0.1890	−0.1535	−0.1556	−0.1486
R	0.5142			
SEM	2.80			
R2	95.5			
	Egg Weight, g			
A1	62.060	65.510	65.770	61.650
B1	−139.60	−175.90	−167.30	−127.30
R	0.89758			
SEM	1.95			
R2	80.5			
	Egg Mass, g			
A	60.430	62.520	63.490	60.940
B	−267,264	−273,987	−243,149	−240,045
C	−0.0858	−0.0384	−0.0431	−0.0740
R	0.6741			
SEM	2.65			
R2	92.3			
	Feed Conversion Ratio, g/g			
A	1.752	1.740	1.740	1.800
B	1,856,646,742	121,069,894	121,069,894	1,135,513,471
C	0.0039	0.0012	0.0012	0.0027
R	0.4019	0.4405	0.4405	0.4019
SEM	0.06			
R2	99.1			
	Body Weight, g			
A	3678.0	4842.0	4615.0	5028.0
B	−2485.0	−3818.0	−3454.0	−3876.0
C	−13.650	−17.910	−16.280	−19.710
R	0.9904			
SEM	103			
R2	52.10			

^1^ Standard error of the mean. ^2^ Coefficient of determination. Models: line plus exponential, A + B × (Rage) + C × age; exponential asymptote, A1 + B1 × (Rage).

**Table 7:** Coefficients of exponential equation for body weight and body composition of laying hens from 19 to 102 weeks old consuming levels of dietary balanced protein (LL or HH), repleted (LH) and depleted feeds (HL).

Parameters	LL	LH	HH	HL
	Ash, %			
A	2.599	2.640	1.960	−3.100
B	2.801	1.500	2.736	7.500
C	0.0169	−0.0385	0.0161	0.0450
R	0.9554	1.0122	0.9723	0.9890
SEM ^1^	0.154			
R2 ^2^	64.70			
	Fat, %			
A	1018	2119	2357	2845
B	−1006	−2110	−2348	−2836
C	1.417	3.045	3.383	4.072
R	1.0013			
SEM	1.65			
R2	51.20			
	Protein, %			
A	15.230	12.700	11.630	10.070
B	3.002	6.525	7.800	9.185
C	−0.0472	−0.1264	−0.1476	−0.1727
R	1.0101			
SEM	0.546			
R2	48.40			

^1^ Standard error of the mean. ^2^ Coefficient of determination. Model: line plus exponential, A + B × (Rage) + C × age.

**Table 8:** Coefficients of exponential equation for egg response of laying hens from 19 to 102 weeks old consuming levels of dietary balanced protein (LL or HH), repleted (LH) and depleted feeds (HL).

Parameters	LL	LH	HH	HL
	Yolk Weight, g			
A1	16.695	17.867	18.189	16.585
B1	−80.000	−47.620	−41.640	−87.100
R	0.88839	0.91377	0.91873	0.88236
SEM ^1^	0.645			
R2 ^2^	86.8			
	Shell Weight, g			
A1	5.990	6.113	6.203	5.951
B1	−0.00154	−0.00083	−0.00095	−0.00145
R	1.0594			
SEM	0.21			
R2	40.2			
	Albumen Weight, g			
A1	40.300	42.450	42.210	39.770
B1	−18.350	−25.020	−23.240	−15.600
R	0.9410			
SEM	1.47			
R2	59.5			
	Shell Strength, kgf			
A1	−6.53	26.3	−5.23	103
B1	13.25	−19.9	11.9	−97
R	0.99677	1.0015	0.99664	1.00032
SEM	0.185			
R2	95			

^1^ Standard error of the mean. ^2^ Coefficient of determination. Model: exponential asymptote, A1 + B1 × (Rage).

## Data Availability

The data presented in this study are available on request from the corresponding author. The data are not publicly available due to privacy.

## Supplementary Materials

The following supporting information can be downloaded at: https://www.mdpi.com/article/10.3390/ani12192567/s1, Table S1: Mean feed intake, feed conversion ratio, and body weight of laying hens from 19 to 102 weeks old in response to levels of dietary balanced protein (LL or HH), repleted (LH) and depleted feeds (HL); Table S2: Mean egg production, egg weight, and egg mass of laying hens from 19 to 102 weeks old in response to levels of dietary balanced protein (LL or HH), repleted (LH) and depleted feeds (HL); Table S3: Body components of laying hens from 19 to 102 weeks old in response to levels of dietary balanced protein (LL or HH), repleted (LH) and depleted feeds (HL); Table S4: Egg components of laying hens from 19 to 102 weeks old in response to levels of dietary balanced protein (LL or HH), repleted (LH) and depleted feeds (HL); Table S5: Shell quality of laying hens from 19 to 102 weeks old in response to levels of dietary balanced protein (LL or HH), repleted (LH) and depleted feeds (HL).
