# Supplementary material for: Response of Laying Hens to Repletion and Depletion in Dietary Balanced Protein
Source: Animals (Basel). 2022 Sep 26;12(19):2567. doi: 10.3390/ani12192567 (PMC9559677; doi:10.3390/ani12192567)
Supplement: Supplementary file 1 [file animals-12-02567-s001.zip › animals-1846888-supplementary.pdf]

Supplementary Tables

Table S1. Mean feed intake, feed conversion ratio, and body weight of laying hens from 19 to 102 weeks old in response to levels of dietary balanced protein (LL or HH), repleted (LH) and depleted feeds (HL).

**Age**	**Feed Intake, g/Bird/Day**						**Feed Conversion Ratio, g/g**						**Body Weight, g/Hen**					
**Weeks [1]**	**LL**	**LH**	**HH**	**HL**	**SEM [2]**	***p*-Value**	**LL**	**LH**	**HH**	**HL**	**SEM**	***p*-Value**	**LL**	**LH**	HH	HL	SEM	***p*-Value**
22	75.9	78.0	80.2	83.6	1.09	0.007	5.47	5.57	3.55	4.08	0.072	<0.0001	1.366	1.353	1.471	1.493	30.7	0.12
26	95.6	96.4	102.1	101.0	1.21	0.004	1.93	1.85	1.84	1.91	0.026	0.05	1.384	1.422	1.504	1.499	37.0	0.20
30	102	103	103	105	1.42	0.77	2.00	1.76	1.77	1.93	0.026	<0.0001	1.374	1.433	1.533	1.510	39.8	0.041
34	105	107	109	108	1.47	0.50	1.95	1.81	1.84	1.97	0.029	<0.001	1.412	1.488	1.564	1.539	38.4	0.049
38	110	110	110	112	1.38	0.86	1.95	1.80	1.76	1.98	0.025	<0.0001	1.449	1.526	1.616	1.614	37.2	0.008
42	106	108	110	108	1.20	0.49	1.87	1.79	1.79	1.86	0.023	0.034	1.466	1.550	1.641	1.621	39.1	0.010
46	109	109	108	110	1.16	0.81	1.94	1.78	1.76	1.93	0.016	<0.0001	1.485	1.577	1.657	1.639	39.7	0.013
50	110	110	110	110	1.57	0.99	1.97	1.81	1.80	1.95	0.022	<0.0001	1.480	1.592	1.681	1.630	44.3	0.004
54	110	111	110	110	1.57	0.89	1.92	1.83	1.80	1.94	0.022	<0.0001	1.472	1.604	1.689	1.641	42.0	0.002
58	110	109	110	111	1.42	0.62	1.93	1.81	1.81	1.93	0.020	<0.0001	1.470	1.625	1.695	1.679	40.2	<0.001
62	113	111	113	112	1.20	0.82	2.01	1.87	1.89	1.96	0.038	0.033	1.445	1.622	1.679	1.681	42.9	0.001
66	115	113	115	115	1.36	0.43	1.87	1.82	1.83	1.89	0.020	0.043	1.479	1.632	1.724	1.703	50.0	<0.001
70	115	113	115	114	1.35	0.84	1.96	1.87	1.85	1.92	0.022	0.002	1.454	1.631	1.703	1.677	46.5	<0.001
74	113	114	115	113	1.84	0.71	1.99	1.84	1.84	2.01	0.030	<0.0001	1.454	1.669	1.743	1.694	47.2	<0.0001
78	109	108	111	112	1.54	0.18	1.98	1.80	1.77	1.97	0.025	<0.0001	1.441	1.655	1.740	1.679	40.9	<0.0001
82	103	109	103	106	1.24	0.14	1.94	1.80	1.76	1.87	0.038	0.006	1.397	1.654	1.696	1.652	38.7	<0.0001
86	106	107	110	105	1.83	0.26	2.14	1.85	1.87	1.99	0.035	<0.0001	1.373	1.649	1.733	1.621	46.3	<0.0001
90	108	109	109	109	2.18	1.00	2.17	1.87	1.79	2.08	0.025	<0.0001	1.357	1.630	1.714	1.632	36.9	<0.0001
94	109	106	107	109	2.44	0.59	2.20	1.83	1.79	2.17	0.035	<0.0001	1.374	1.589	1.672	1.591	42.8	<0.001
98	114	112	111	112	2.02	0.65	2.22	1.93	1.90	2.14	0.034	<0.0001	1.398	1.565	1.675	1.619	37.3	0.001
102	111	113	110	113	2.26	0.72	2.20	1.95	1.98	2.11	0.035	<0.0001	1.341	1.621	1.689	1.502	46.8	0.002

[1] Every 4 weeks from 19 to 102 weeks of age. [2] SEM: Standard error of the mean.

Table S2. Mean egg production, egg weight, and egg mass of laying hens from 19 to 102 weeks old in response to levels of dietary balanced protein (LL or HH), repleted (LH) and depleted feeds (HL).

Age	Egg Production, %						Egg Weight, g						Egg Mass, g					
Weeks [1]	LL	LH	HH	HL	SEM [2]	*p*-Value	LL	LH	HH	HL	SEM	*p*-Value	LL	LH	HH	HL	SEM	*p*-Value
22	27.0	29.4	45.8	37.3	2.071	<0.0001	48.4	48.2	49.4	48.7	0.558	0.70	13.1	15.0	20.9	18.1	1.101	<0.0001
26	92.1	93.2	96.8	95.6	0.970	0.06	54.7	56.1	57.4	55.4	0.451	0.043	49.7	52.3	55.6	52.9	0.750	0.001
30	90.4	97.8	97.5	95.0	0.959	<0.001	56.9	59.9	59.8	57.2	0.561	0.003	51.4	58.6	58.4	54.3	0.859	<0.0001
34	92.6	97.3	97.4	93.7	1.078	0.023	58.6	61.0	60.9	58.6	0.566	0.010	54.3	59.4	60.4	54.9	0.900	<0.0001
38	94.4	97.5	98.0	94.5	0.721	0.12	60.0	62.6	62.4	59.9	0.552	0.001	56.7	61.0	61.9	56.6	0.828	<0.001
42	94.6	95.9	97.9	96.7	0.774	0.38	60.1	62.8	62.6	60.2	0.499	0.004	56.9	60.3	61.3	58.1	0.662	0.012
46	93.8	96.9	96.9	95.8	0.708	0.37	59.8	63.1	63.4	59.6	0.610	<0.0001	56.1	61.1	61.4	57.1	0.691	<0.001
50	91.9	96.5	95.8	94.2	0.918	0.10	60.5	63.1	64.0	60.1	0.642	<0.0001	55.6	60.9	60.6	56.6	0.903	<0.001
54	92.8	95.2	97.3	93.6	0.961	0.16	61.6	63.9	64.3	61.6	0.639	0.003	57.2	60.8	61.6	56.8	1.024	0.001
58	92.1	93.3	94.9	93.8	1.068	0.49	61.9	63.8	63.6	61.6	0.642	0.007	56.3	59.6	60.8	57.8	0.988	0.019
62	88.7	92.3	92.2	91.8	1.783	0.20	63.3	64.4	65.1	62.8	0.642	0.06	56.1	59.4	60.5	57.7	1.188	0.035
66	95.1	95.8	94.9	96.5	0.739	0.96	64.6	65.3	66.3	64.0	0.637	0.07	61.4	61.9	62.9	60.8	0.922	0.56
70	92.5	92.7	93.8	95.8	1.377	0.50	62.7	65.4	66.1	62.0	0.637	<0.0001	59.1	60.5	62.0	58.4	0.846	0.06
74	91.4	94.1	93.9	91.4	1.497	0.32	62.1	66.2	66.5	61.3	0.661	<0.0001	56.8	62.2	62.4	56.0	1.073	<0.0001
78	88.8	91.4	94.1	93.4	1.637	0.043	62.0	66.0	66.4	62.2	0.596	<0.0001	55.0	60.3	62.5	57.1	1.095	<0.0001
82	86.4	91.9	89.6	92.6	1.579	0.008	61.8	66.1	66.0	61.3	0.698	<0.0001	53.5	60.7	59.1	56.7	1.033	<0.0001
86	82.9	88.6	89.4	89.0	1.697	0.002	60.7	65.7	66.0	60.6	0.717	<0.0001	49.5	58.1	59.2	53.8	1.089	<0.0001
90	80.6	87.7	91.6	86.3	1.802	<0.0001	61.2	66.4	65.4	60.8	0.638	<0.0001	49.4	58.2	61.5	52.4	1.213	<0.0001
94	81.2	89.2	90.0	82.8	1.711	<0.0001	61.3	66.3	67.5	60.8	0.692	<0.0001	49.8	58.1	60.4	50.3	1.376	<0.0001
98	82.3	87.7	88.2	86.6	1.869	0.05	61.7	66.2	66.2	60.9	0.724	<0.0001	51.7	58.0	57.3	52.8	1.232	<0.0001
102	81.0	86.8	86.5	87.2	2.061	0.008	62.4	66.7	66.4	61.5	0.645	<0.0001	50.7	57.9	56.1	52.6	1.460	<0.0001

[1] Every 4 weeks from 19 to 102 weeks of age. [2] SEM: Standard error of the mean.

Table S3. Body components of laying hens from 19 to 102 weeks old in response to levels of dietary balanced protein (LL or HH), repleted (LH) and depleted feeds (HL).

Age	Ash, %						Fat, %						Protein, %					
Weeks [1]	LL	LH	HH	HL	SEM [2]	*p*-Value	LL	LH	HH	HL	SEM	*p*-Value	LL	LH	HH	HL	SEM	*p*-Value
22	4.05	3.83	3.84	3.82	0.061	0.51	13.1	12.7	13.2	14.3	0.445	0.54	18.4	18.5	18.4	17.9	0.144	0.43
26	3.84	3.54	3.62	3.76	0.081	0.13	15.2	16.2	15.5	15.8	0.863	0.92	17.3	17.1	17.5	17.3	0.332	0.88
30	3.87	3.75	3.61	3.68	0.069	0.07	13.4	14.0	14.8	15.6	0.619	0.18	18.1	18.0	17.9	17.4	0.242	0.22
34	3.77	3.58	3.54	3.62	0.068	0.10	14.2	15.5	16.0	16.5	0.730	0.26	17.9	17.5	17.5	17.2	0.270	0.31
38	3.74	3.50	3.51	3.54	0.064	0.05	14.6	15.8	16.5	18.1	0.805	0.021	17.8	17.5	17.3	16.6	0.320	0.020
42	3.74	3.52	3.47	3.56	0.064	0.024	14.4	16.0	16.1	17.0	0.740	0.08	17.9	17.5	17.6	17.0	0.280	0.15
46	3.73	3.50	3.50	3.57	0.068	0.07	15.3	16.2	16.8	17.3	0.796	0.32	17.5	17.4	17.3	17.0	0.296	0.54
50	3.66	3.51	3.42	3.49	0.063	0.013	15.1	17.2	17.8	19.2	0.723	0.001	17.6	17.0	16.9	16.3	0.239	0.011
54	3.78	3.43	3.47	3.59	0.066	0.002	15.3	17.9	18.1	18.8	0.751	0.008	17.5	16.7	16.8	16.3	0.260	0.020
58	3.68	3.48	3.46	3.48	0.057	0.006	13.2	17.0	16.1	17.6	0.652	<.0001	18.5	17.2	17.7	16.9	0.224	<.001
62	3.67	3.49	3.43	3.43	0.063	0.008	13.6	16.3	16.3	17.7	0.808	0.002	18.3	17.5	17.6	17.0	0.266	0.006
66	3.78	3.41	3.48	3.49	0.063	<.001	14.2	16.7	17.3	19.0	0.771	<.001	18.0	16.9	17.2	16.4	0.307	<.001
70	3.94	3.45	3.39	3.65	0.076	<.0001	13.9	16.1	19.0	18.5	0.840	<.0001	18.0	17.6	16.8	16.5	0.288	<.0001
74	3.99	3.45	3.44	3.59	0.075	<.0001	14.4	18.1	18.2	19.2	0.815	<.0001	17.7	16.7	16.8	15.9	0.263	0.001
78	3.98	3.40	3.45	3.54	0.073	<.0001	13.5	17.8	18.4	18.8	0.851	<.0001	18.1	16.9	16.7	16.4	0.295	<.0001
82	3.96	3.52	3.56	3.61	0.084	<.001	13.3	17.0	17.2	17.4	0.745	<.0001	18.2	17.2	17.1	16.9	0.262	0.003
86	4.26	3.63	3.58	3.80	0.088	<.0001	12.2	16.3	16.5	16.0	0.656	<.0001	18.5	17.4	17.4	17.3	0.228	0.007
90	4.20	3.61	3.70	3.77	0.099	<.0001	12.9	16.7	15.6	15.9	0.806	0.008	18.3	17.2	17.7	17.4	0.276	0.06
94	4.36	3.77	3.75	3.83	0.081	<.0001	11.8	15.1	14.6	15.3	0.643	0.002	18.7	17.8	18.1	17.6	0.229	0.035
98	4.29	3.82	3.70	3.87	0.071	<.0001	11.7	14.6	14.7	14.1	0.533	0.029	18.8	18.0	18.1	18.1	0.200	0.15
102	4.26	3.82	3.70	4.02	0.099	0.002	11.9	14.5	15.3	14.6	0.870	0.05	18.6	17.9	18.3	17.7	0.296	0.07

[1] Every 4 weeks from 19 to 102 weeks of age.[2] SEM: Standard error of the mean.

Table S4. Egg components of laying hens from 19 to 102 weeks old in response to levels of dietary balanced protein (LL or HH), repleted (LH) and depleted feeds (HL).

**Age**	**Yolk, g**						**Shell, g**						**Albumen, g**					
**Weeks** [1]	**LL**	**LH**	**HH**	**HL**	**SEM** [2]	***p*-Value**	**LL**	**LH**	**HH**	**HL**	**SEM**	***p*-Value**	**LL**	**LH**	**HH**	**HL**	**SEM**	***p*-Value**
22	10.7	10.8	11.2	10.9	0.137	0.57	5.65	5.69	5.87	5.59	0.067	0.12	34.9	34.6	35.0	35.0	0.345	0.96
26	13.2	14.0	14.4	13.5	0.196	0.009	5.81	6.05	6.19	5.87	0.073	0.004	36.4	38.6	38.6	36.9	0.420	0.006
30	14.3	15.1	15.3	14.7	0.218	0.012	5.92	6.14	6.09	5.84	0.075	0.049	38.2	39.3	39.2	38.0	0.427	0.19
34	15.2	15.5	15.7	15.3	0.205	0.42	6.07	6.14	6.27	5.87	0.088	0.005	38.5	39.8	39.8	38.6	0.487	0.09
38	15.9	16.2	16.4	15.4	0.282	0.06	6.21	6.23	6.33	6.11	0.082	0.29	38.5	40.0	39.6	38.2	0.524	0.045
42	16.0	16.7	17.0	15.7	0.239	0.001	6.08	6.11	6.15	5.95	0.059	0.26	39.4	40.4	40.5	37.9	0.563	0.005
46	15.9	16.8	17.0	16.1	0.274	0.008	6.05	6.19	6.26	6.05	0.073	0.15	38.2	40.6	40.2	38.0	0.570	<0.001
50	16.3	17.3	17.2	16.2	0.246	0.001	5.88	6.08	6.10	5.84	0.080	0.019	38.2	40.8	40.4	38.2	0.544	<0.0001
54	16.8	17.0	17.5	17.3	0.239	0.29	6.08	6.13	6.22	6.14	0.076	0.61	39.7	40.6	40.8	39.6	0.551	0.20
58	16.5	17.3	17.3	16.6	0.251	0.030	6.00	6.16	6.20	6.00	0.076	0.13	39.6	40.6	40.8	39.0	0.519	0.07
62	17.3	17.5	17.9	17.5	0.250	0.42	6.03	6.07	6.19	6.02	0.098	0.48	41.1	41.3	41.3	41.2	0.546	0.99
66	17.6	17.8	18.4	17.6	0.247	0.045	6.16	6.09	6.31	6.18	0.078	0.25	41.3	41.4	41.6	40.8	0.500	0.77
70	16.8	17.7	18.4	16.6	0.287	<0.0001	5.94	6.16	6.31	5.96	0.066	<.001	40.3	42.3	41.8	39.4	0.583	0.002
74	17.0	18.4	18.3	16.8	0.259	<0.0001	5.89	6.17	6.12	5.88	0.073	0.011	39.7	42.2	41.7	39.8	0.535	0.002
78	16.5	17.8	18.5	16.7	0.240	<0.0001	5.75	5.98	6.12	5.78	0.085	0.013	39.8	41.8	42.9	39.8	0.578	<0.0001
82	16.7	18.1	18.3	16.3	0.243	<0.0001	5.60	6.04	6.05	5.62	0.072	<0.0001	39.5	43.1	41.9	38.9	0.643	<0.0001
86	16.8	17.6	18.3	16.1	0.287	<0.0001	5.62	5.90	6.05	5.49	0.092	<0.0001	39.6	41.4	42.2	38.6	0.564	<0.0001
90	16.1	17.9	18.4	16.1	0.292	<0.0001	5.67	6.04	6.11	5.56	0.094	<0.0001	39.8	42.9	43.0	39.5	0.614	<0.0001
94	16.0	18.0	17.9	16.3	0.276	<0.0001	5.61	5.90	5.88	5.59	0.100	0.027	39.7	43.1	42.2	39.2	0.643	<0.0001
98	16.1	18.0	18.1	16.2	0.283	<0.0001	5.47	5.86	5.91	5.71	0.104	0.007	40.6	43.8	42.9	39.4	0.680	<0.0001
102	17.0	18.1	18.1	16.1	0.298	<0.0001	5.63	5.80	5.86	5.45	0.104	0.036	41.2	42.7	41.9	40.7	0.678	0.23

[1] Every 4 weeks from 19 to 102 weeks of age. [2] SEM: Standard error of the mean.

Table S5. Shell quality of laying hens from 19 to 102 weeks old in response to levels of dietary balanced protein (LL or HH), repleted (LH) and depleted feeds (HL).

Age Weeks [1]	Shell Strength, kgf						Shell Thickness, mm					
	LL	LH	HH	HL	SEM [2]	*p*-Value	LL	LH	HH	HL	SEM	*p*-Value
22	5.81	5.77	5.77	5.66	0.077	0.74	0.448	0.437	0.450	0.439	0.005	0.32
26	5.59	5.58	5.71	5.59	0.082	0.76	0.404	0.405	0.414	0.408	0.003	0.21
30	5.28	5.42	5.38	5.16	0.089	0.30	0.395	0.399	0.393	0.389	0.004	0.56
34	5.38	5.18	5.36	4.77	0.097	<.001	0.404	0.401	0.400	0.388	0.005	0.10
38	5.33	5.25	5.29	5.22	0.109	0.90	0.402	0.401	0.406	0.403	0.004	0.83
42	5.07	5.14	5.13	5.08	0.089	0.95	0.394	0.386	0.394	0.388	0.003	0.31
46	5.01	5.03	5.14	5.02	0.103	0.81	0.398	0.397	0.396	0.399	0.004	0.96
50	4.65	4.89	5.00	4.63	0.119	0.029	0.403	0.392	0.400	0.395	0.004	0.28
54	4.67	4.85	4.73	4.74	0.103	0.67	0.384	0.384	0.387	0.387	0.005	0.93
58	4.70	4.77	4.56	4.64	0.115	0.56	0.385	0.382	0.388	0.387	0.004	0.75
62	4.46	4.41	4.57	4.29	0.096	0.27	0.386	0.389	0.390	0.390	0.005	0.91
66	4.14	4.23	4.34	4.12	0.102	0.45	0.387	0.371	0.379	0.384	0.003	0.017
70	3.98	4.12	4.12	4.18	0.104	0.67	0.368	0.368	0.378	0.374	0.004	0.12
74	3.79	4.04	3.80	3.88	0.111	0.29	0.368	0.370	0.370	0.371	0.004	0.96
78	3.76	3.86	3.88	4.00	0.102	0.61	0.357	0.359	0.363	0.356	0.004	0.59
82	3.33	3.64	3.65	3.43	0.101	0.07	0.353	0.361	0.360	0.352	0.003	0.11
86	3.30	3.61	3.47	3.39	0.110	0.17	0.353	0.355	0.357	0.349	0.005	0.60
90	3.35	3.58	3.62	3.19	0.106	0.014	0.349	0.356	0.358	0.353	0.004	0.38
94	3.29	3.36	3.77	3.41	0.115	0.013	0.353	0.352	0.355	0.356	0.005	0.94
98	3.24	3.20	3.31	3.41	0.100	0.49	0.361	0.368	0.370	0.376	0.005	0.16
102	3.17	3.22	3.28	3.07	0.094	0.50	0.341	0.345	0.345	0.343	0.004	0.90

[1] Every 4 weeks from 19 to 102 weeks of age. [2] SEM: Standard error of the mean.
